# A randomized, controlled comparative study of the wrinkle reduction benefits of a cosmetic niacinamide/peptide/retinyl propionate product regimen vs. a prescription 0·02% tretinoin product regimen

**DOI:** 10.1111/j.1365-2133.2009.09436.x

**Published:** 2010-03

**Authors:** JJJ Fu, GG Hillebrand, P Raleigh, J Li, MJ Marmor, V Bertucci, PE Grimes, SH Mandy, MI Perez, SH Weinkle, JR Kaczvinsky

**Affiliations:** Private practiceMason, OH, U.S.A.; * The Procter & Gamble Company11510 Reed Hartman Highway, Cincinnati, OH 45241-9974, U.S.A.; † University of TorontoToronto, ON, Canada; ‡ UCLA Medical CenterLos Angeles, CA, U.S.A.; § University of MiamiMiami, FL, U.S.A.; ¶ Columbia UniversityNew York, NY, U.S.A.; ** Private practiceBradenton, FL, U.S.A.

**Keywords:** anti-ageing, niacinamide, retinyl propionate, skin care, tretinoin, wrinkles

## Abstract

**Background:**

Tretinoin is considered the benchmark prescription topical therapy for improving fine facial wrinkles, but skin tolerance issues can affect patient compliance. In contrast, cosmetic antiwrinkle products are well tolerated but are generally presumed to be less efficacious than tretinoin.

**Objectives:**

To compare the efficacy of a cosmetic moisturizer regimen vs. a prescription regimen with 0·02% tretinoin for improving the appearance of facial wrinkles.

**Methods:**

An 8-week, randomized, parallel-group study was conducted in 196 women with moderate to moderately severe periorbital wrinkles. Following 2 weeks washout, subjects on the cosmetic regimen (*n*=99) used a sun protection factor (SPF) 30 moisturizing lotion containing 5% niacinamide, peptides and antioxidants, a moisturizing cream containing niacinamide and peptides, and a targeted wrinkle product containing niacinamide, peptides and 0·3% retinyl propionate. Subjects on the prescription regimen (*n*=97) used 0·02% tretinoin plus moisturizing SPF 30 sunscreen. Subject cohorts (*n*=25) continued treatment for an additional 16 weeks. Changes in facial wrinkling were assessed by both expert grading and image analysis of digital images of subjects’ faces and by self-assessment questionnaire. Product tolerance was assessed via clinical erythema and dryness grading, subject self-assessment, and determinations of skin barrier integrity (transepidermal water loss) and stratum corneum protein changes.

**Results:**

The cosmetic regimen significantly improved wrinkle appearance after 8 weeks relative to tretinoin, with comparable benefits after 24 weeks. The cosmetic regimen was significantly better tolerated than tretinoin through 8 weeks by all measures.

**Conclusions:**

An appropriately designed cosmetic regimen can improve facial wrinkle appearance comparably with the benchmark prescription treatment, with improved tolerability.

Topical tretinoin is considered a benchmark treatment for the mitigation of fine facial wrinkles.^1^–^3^ Use of tretinoin cream (0·02%) has demonstrated a benefit after 24 weeks of treatment.^1^ However, there are several side-effects (e.g. erythema and desquamation) associated with tretinoin use, especially during the first few weeks, which may cause patients to discontinue use.^4^ One study showed that concomitant use of a niacinamide-containing moisturizer with tretinoin therapy augmented the response to tretinoin and improved the stratum corneum, decreasing tretinoin-associated side-effects.^5^

In contrast to the irritancy potential of retinoid therapies, cosmetic and cosmeceutical antiwrinkle products are generally well tolerated by the skin and are pleasant for patients to use. While there are few published reports of direct comparative studies,^6^ it is generally presumed that such products do not have clinical efficacy comparable with that of prescription topical therapies such as tretinoin.

To determine whether significant wrinkle reduction efficacy comparable with that of a prescription could be achieved using cosmetic products, a treatment regimen was developed containing niacinamide, the palmitoyl peptides palmitoyl-lysine-threonine and palmitoyl-lysine-threonine-threonine-lysine-serine (Pal-KT and Pal-KTTKS, respectively),^7^–^12^ and a less irritating retinyl ester commonly used in over-the-counter and cosmetic products (retinyl propionate).^13^,^14^ A study was conducted to compare the efficacy of this cosmetic niacinamide/peptide/retinyl propionate (NPP) product regimen vs. a prescription 0·02% tretinoin product regimen for improving the appearance of facial fine lines and wrinkles. Product regimen tolerability was also assessed.

## Materials and methods

### Study design and subjects

**Fig 1:**
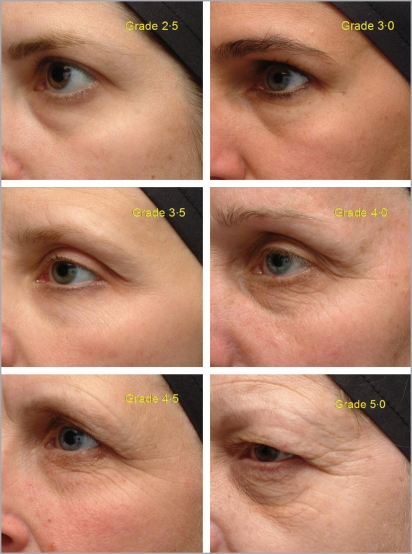
Images illustrating the range of wrinkle severity suitable for study inclusion. Severity was graded on a 0–5 scale (at 0·5-grade increments). Enrolled subjects had grades between 3·5 and 4·5, inclusive, on each side of their face. Images depicting the portion of the scale including and bracketing the acceptable grades are shown.

An 8-week, randomized, parallel-group facial appearance study was performed in Cincinnati, OH, U.S.A. from February to August 2008 in 196 women aged 40–65 years who were neither pregnant nor lactating. Eligible subjects had Fitzpatrick skin types I–III and moderate to moderately severe periorbital wrinkles on both sides of their face. Wrinkle severity was assessed using a six-point ordinal photonumeric scale (Fig.1). Subjects preconditioned their face for 2 weeks using a mild facial cleanser (Olay® Foaming Face Wash; Procter & Gamble Company, Cincinnati, OH, U.S.A.) *ad libidum* and a facial moisturizer (Olay® Complete All Day Moisture Lotion Sensitive Skin SPF 15; Procter & Gamble Company) twice daily in place of their regular cleansing and moisturizing products. After the preconditioning period, subjects were randomly assigned to either the NPP regimen or the tretinoin regimen for 8 weeks of treatment. A computer-generated randomization list was drawn up by the study statistician and was given to the study site for treatment allocation. Prior to study start, 25 self-selected subjects (cohort) on each product regimen agreed to continue treatment for an additional 16 weeks. Written informed consent for participation in this study was obtained from all subjects in accordance with the Helsinki II declaration, and the protocol was approved by an Institutional Review Board (Schulman Associates IRB, Inc., Cincinnati, OH, U.S.A.).

### Study products

Subjects were instructed to dose both product regimens as per the manufacturer’s package instructions or, when appropriate, investigator guidance. Subjects assigned to the NPP regimen used a daytime sun protection factor (SPF) 30 lotion (Olay® Professional Pro-X Age Repair Lotion SPF 30; Procter & Gamble Company) in the morning, and a night cream (Olay® Professional Pro-X Wrinkle Smoothing Cream; Procter & Gamble Company) in the evening, both over the entire face. In addition, these subjects applied a wrinkle treatment (Olay® Professional Pro-X Deep Wrinkle Treatment; Procter & Gamble Company) twice daily, that is, prior to the daytime SPF 30 lotion and the night cream, on areas of concern, as determined by the subject. Table 1 shows the ingredient list for the test products. All the test products in the NPP regimen contained niacinamide, the peptides Pal-KT and Pal-KTTKS, and carnosine in a moisturizing base. The daytime SPF 30 lotion also contained a broad-spectrum sunscreen (homosalate, avobenzone, ensulizole, octocrylene) and vitamins C and E (sodium ascorbyl phosphate and tocopheryl acetate). The wrinkle treatment contained 0·3% retinyl propionate. The subjects randomized to the tretinoin regimen applied 0·02% tretinoin in an emollient base (Renova®; Neutrogena Corporation, Los Angeles, CA, U.S.A.)^15^ to the entire face every other evening and a manufacturer-recommended SPF 30 moisturizing sunscreen (Neutrogena Healthy Defense® Daily Moisturizer SPF 30; Johnson and Johnson) every morning for the first 2 weeks; after the initial 2-week period, subjects used the 0·02% tretinoin every evening. Subjects of childbearing age in the tretinoin regimen group had monthly urine pregnancy tests during the study. To assess compliance, the products were weighed at baseline and at each visit. Subjects and study personnel dispensing product were not blinded to treatment.

**Table 1:** Ingredients for niacinamide/peptide/retinyl propionate (NPP) regimen products (in a moisturizing base)

Ingredient	Daytime SPF 30 lotion	Night cream	Wrinkle treatment
Niacinamide	X	X	X
Pal-KTTKS	X	X	X
Pal-KT	X	X	X
Carnosine	X	X	X
Unique ingredients	SPF 30, vitamin C, vitamin E	–	Retinyl propionate

Pal-KTTKS, palmitoyl-lysine-threonine-threonine-lysine-serine; Pal-KT, palmitoyl-lysine-threonine; SPF, sun protection factor.

### Assessments

Improvement in the appearance of fine lines and wrinkles was measured by expert visual grading of high-resolution digital images using the Rapid Evaluation of Anti-aging Leads (REAL 3.0) system^16^ taken at baseline and at 8 and (in the cohort) 24 weeks. Three trained expert graders independently assessed changes in the appearance of fine lines and wrinkles around the eyes by comparing identified baseline and post-treatment images side-by-side using a ± eight-point ordinal scale. The expert graders and other assessors were blinded to the treatments. Dermatologists on the study advisory panel evaluated a set of representative study images for concurrence with the outcomes observed in expert grading. Images of the infraorbital/crow’s feet areas were also taken at baseline and at 8 and 24 weeks with the commercially available VISIA CR imaging system (Canfield Scientific, Fairfield, NJ, U.S.A.) and analysed for changes in periorbital wrinkle area fraction (wrinkle area divided by assessment area).

In addition, each subject completed a self-assessment questionnaire containing 14 questions evaluating nine efficacy attributes (fine lines and wrinkles around the eyes, evenness of skin tone, red blotchiness, age spots, evenness of skin texture, skin firmness, skin radiance, skin hydration, and overall appearance) using a 10-point continuous scale at baseline and at 2, 4, 6 and 8 weeks in the entire study population and additionally at 16 and 24 weeks in the cohort.

Subject tolerability to each regimen was measured by expert clinical grading of erythema and dryness using a six-point scale and by a subject self-assessment questionnaire using a four-point scale at baseline and at 2, 4, 6 and 8 weeks in the entire population and additionally at 16 and 24 weeks in the cohort. Skin barrier integrity was measured by transepidermal water loss (TEWL) at two sites on the face using a Vapometer SWL-2 (Delfin Technologies, Ltd, Kuopio, Finland).

D-Squame® tape strips (CuDerm Corporation, Dallas, TX, U.S.A.) from the right cheek were collected at baseline and at 8 and 24 weeks, and analysed for the amount of soluble protein, human serum albumin (HSA), involucrin, keratin 6, keratins 1, 10 and 11, fibronectin and cortisol via immunoassay (SkinMAP; Millipore, St Charles, MO, U.S.A.).^17^ Levels of keratin 6, fibronectin and cortisol from over 90% of the subjects were below detection limits; therefore, reliable comparisons could not be made for these three analytes.

### Statistical analysis

The primary analysis variable was expert visual grading for the change in appearance of fine lines and wrinkles at 8 weeks. For post-treatment analysis, expert visual grades for the change in appearance of fine lines and wrinkles, change from baseline for wrinkle area fraction, erythema, dryness, self-assessment efficacy questions, and protein analytes from D-Squame® tape strips (normalized to total protein and log transformed) were all analysed, separately, via an analysis of covariance model with age and baseline wrinkle grade as covariates using SAS version 9.1 software (SAS Institute, Cary, NC, U.S.A.). Self-assessment tolerability questions were analysed using χ^2^ tests. Results were considered significant if *P*<0·05 (two-sided). Ninety-three subjects would permit detection of a mean wrinkle grade difference of 0·28 at 95% significance (two-sided) with an assumed SD of 0·95 and 80% power. Assuming a 5% drop-out rate, 98 subjects for each group (196 in total) were required.

## Results

### Subject accountability and baseline characteristics

Of the 97 tretinoin regimen subjects and 99 NPP regimen subjects who started the study, 93 and 97 subjects, respectively, completed the study. One subject in the tretinoin regimen group withdrew due to redness and dryness; three subjects in the tretinoin regimen group and two in the NPP regimen group were withdrawn due to noncompliance. Five subjects (three in the tretinoin regimen group; two in the NPP regimen group) were excluded from the REAL image analysis due to image quality issues (facial expressions, etc.). Table 2 shows the demographics and baseline values of the two balanced groups.

**Table 2:** Demographics and baseline values of key parameters

	Tretinoin regimen	NPP regimen	*P*-value
Age (years), mean ± SD (range)	53·1 ± 6·2 (40–65) (*n*=97)	52·2 ± 6·2 (40–64) (*n*=99)	0·05
Wrinkle area fraction (VISIA CR image analysis), mean ± SEM	0·623 ± 0·019 (*n*=93)	0·633 ± 0·019 (*n*=97)	0·70
Erythema, mean ± SEM	0·972 ± 0·067 (*n*=97)	0·929 ± 0·066 (*n*=99)	0·65
Dryness, mean ± SEM	0·031 ± 0·016 (*n*=97)	0·038 ± 0·016 (*n*=99)	0·76
TEWL (g m^−2^ h^−1^), mean ± SEM	13·225 ± 0·335 (*n*=95)	12·868 ± 0·328 (*n*=99)	0·45

NPP, niacinamide/peptide/retinyl propionate; TEWL, transepidermal water loss.

### Eight-week results

**Fig 2:**
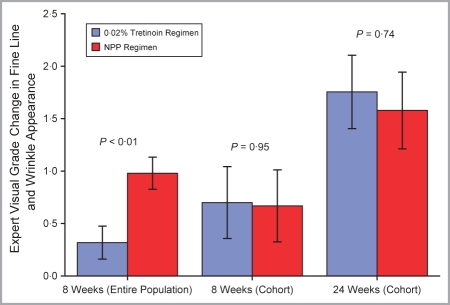
Improvement in the appearance of periorbital wrinkles for the full study population and 24-week cohort. Changes were determined by expert visual comparison of subject images before and after treatment, using a ± eight-point scale. Error bars represent SEM. Entire population at 8 weeks: *n*=90 in the tretinoin regimen group; *n*=95 in the niacinamide/peptide/retinyl propionate (NPP) regimen group. Cohort at 8 weeks: *n*=25 in each group. Cohort at 24 weeks: *n*=25 in the tretinoin regimen group; *n*=23 in the NPP regimen group.

**Fig 3:**
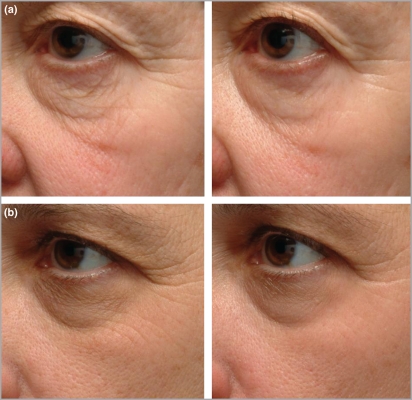
(a) Niacinamide/peptide/retinyl propionate regimen before and after 8 weeks of treatment. (b) Tretinoin regimen before and after 8 weeks of treatment.

After 8 weeks of treatment, the appearance of facial fine lines and wrinkles improved in both groups as measured by expert visual grading of the REAL images (*P*=0·05, tretinoin regimen; *P*<0·01, NPP regimen). The NPP regimen gave significantly greater improvement than the tretinoin regimen (*P*<0·01; Fig. 2). A significantly higher percentage of subjects on the NPP regimen (58%) was judged to look better after 8 weeks of treatment, defined as being given the minimum positive grade of +1 on each side of their face by at least two of the three graders, compared with the tretinoin regimen (41%, *P*=0·03). Also, a significantly higher percentage of subjects on the NPP regimen responded substantially to treatment (mean grade ≥ 2) as compared with the tretinoin regimen (28% vs. 13%, *P*=0·02). For perspective, a +2 grade would indicate visible improvement in at least one to three small lines and/or 15–25% of areas of crepiness or cross-hatching. Examples of subjects showing substantial responses to each treatment regimen are shown in Figure 3.

**Fig 4:**
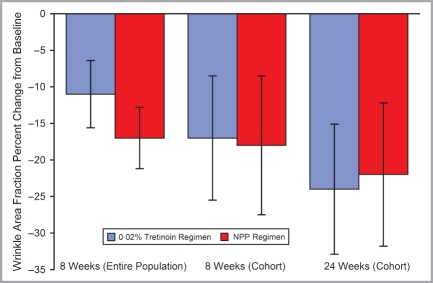
Reductions in wrinkle area (as a fraction of the facial area of interest) for the full study population and 24-week cohort. Wrinkle area was determined by computer image analysis. Error bars indicate 95% confidence interval. Entire population at 8 weeks: *n*=92 in the tretinoin regimen group; *n*=95 in the niacinamide/peptide/retinyl propionate (NPP) regimen group. Cohort at 8 weeks: *n*=25 in the tretinoin regimen group; *n*=24 in the NPP regimen group. Cohort at 24 weeks: *n*=25 in the tretinoin regimen group; *n*=22 in the NPP regimen group.

As determined by analysis of the VISIA CR images, both product regimens also significantly reduced periorbital wrinkle area fraction at week 8 relative to baseline (*P*<0·01 for both). The mean percentage reduction from baseline in wrinkle area fraction was greater for the NPP regimen group vs. the tretinoin regimen group (17% vs. 11%, *P*=0·06, Fig. 4). The change from baseline of relevant efficacy self-assessment questions at 4 and 8 weeks is shown in Table 3. Both groups noticed significant improvement from baseline in overall skin appearance, fine lines and wrinkles, and eye lines and wrinkles at both time points. After 4 weeks, subjects in the NPP regimen group noticed a significant improvement from baseline in overall skin feel and uneven skin texture, and both groups noticed a significant improvement in these assessments at 8 weeks. By 8 weeks, both groups noticed improvement from baseline in deep wrinkles. The self-assessed improvements in overall skin appearance, overall skin feel, and eye lines and wrinkles were significantly greater for the NPP regimen group than for the tretinoin group after both 4 and 8 weeks, and for uneven skin texture after 4 weeks.

**Table 3:** Relevant efficacy assessment questions from the self-assessment questionnaire (change from baseline; full study population)

	Week 4		Week 8	
Question	Tretinoin regimen	NPP regimen	Tretinoin regimen	NPP regimen
Overall skin appearance	0·287*	1·211*†	1·296*	1·792*†
Overall skin feel	−0·150	1·360*†	0·759*	1·889*†
Fine lines/wrinkles	−0·625*	−0·899*	−1·110*	−1·466*
Eye lines/wrinkles	−0·651*	−1·206*†	−1·337*	−1·975*†
Deep wrinkles	−0·233	−0·218	−0·562*	−0·563*
Uneven skin texture	0·300	−0·528*†	−0·506*	−0·810*

NPP, niacinamide/peptide/retinyl propionate.

* Statistically significantly different from baseline at *P*<0·05.

† Statistically significantly different from the tretinoin regimen at *P*<0·05. Attributes were graded by subjects on a 10-point continuous scale at baseline and subsequent assessment periods. Changes in grade were computed and compared, with positive numbers meaning improvement for skin appearance and feel and negative numbers (i.e. having less) representing improvement for the other attributes.

**Fig 5:**
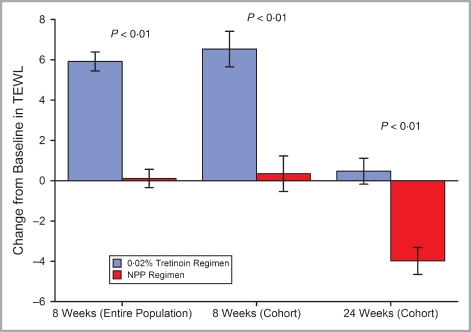
Changes in facial skin transepidermal water loss (TEWL) for the full study population and 24-week cohort. At 8 weeks, *n*=91 for the full population tretinoin group and *n*=97 for the full population niacinamide/peptide/retinyl propionate (NPP) regimen group; *n*=25 for both cohorts. At 24 weeks, *n*=25 for the tretinoin regimen cohort and *n*=23 for the NPP regimen cohort.

After 8 weeks of treatment, the stratum corneum barrier in the tretinoin regimen group was significantly compromised, with TEWL increasing by 5·9 g m^−2^ h^−1^ from baseline (Fig. 5). TEWL values in the NPP regimen group remained essentially unchanged from baseline. There was a significant difference in change in TEWL between the two groups at 8 weeks (*P*<0·01).

**Fig 6:**
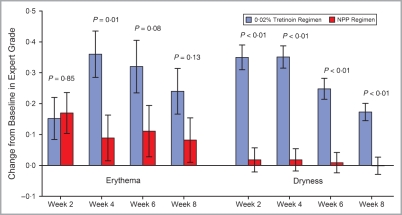
Erythema and skin dryness during the initial 8 weeks of treatment (full study population). Both erythema and clinical dryness were determined on a 0–6 scale. For the tretinoin regimen, *n*=95 (week 2), *n*=94 (week 4) and *n*=93 (weeks 6 and 8). For the niacinamide/peptide/retinyl propionate (NPP) regimen, *n*=99 (week 2) and *n*=97 (weeks 4, 6 and 8).

After 2 weeks of treatment, both product regimen groups exhibited a significant increase in erythema from baseline (Fig. 6). After 4 weeks of treatment, erythema continued to be significantly increased in the tretinoin regimen group and was significantly higher compared with the NPP regimen group (*P*=0·01). Erythema in the NPP regimen group was lower at 4, 6 and 8 weeks compared with 2 weeks and was no longer statistically significantly different from baseline. At 6 and 8 weeks, erythema did decrease in the tretinoin regimen but remained significantly higher than at baseline.

At 2, 4, 6 and 8 weeks, the tretinoin regimen group showed significantly more skin dryness from baseline (Fig. 6). There was no significant change from baseline in skin dryness in the NPP regimen group at 2, 4, 6 and 8 weeks. In addition, the skin of subjects in the tretinoin regimen group was significantly drier than the skin of subjects in the NPP regimen group at 2, 4, 6 and 8 weeks (*P*<0·01 for all).

The results of the subjects’ self-assessments of product tolerability were consistent with the expert erythema and dryness results (data not shown). Compared with the NPP regimen group, after 2 weeks of treatment significantly more subjects in the tretinoin regimen group experienced itchiness, redness/rash and peeling/flaking, which is consistent with known side-effects of tretinoin.^4^ Symptoms continued in the tretinoin regimen group for the entire 8-week study with a peak between 4 and 6 weeks. Subjects in the NPP regimen group who reported any irritation at any time point generally reported experiencing it only a ‘slight amount’.

There were significant differences between the product regimens for each of the four stratum corneum protein analytes after 8 weeks of treatment (Table 4). Soluble protein, HSA and involucrin levels were significantly increased from baseline in the tretinoin regimen group. In contrast, in the NPP regimen group, soluble protein and HSA decreased from baseline, and involucrin was essentially unchanged from baseline. Keratins 1, 10 and 11 significantly decreased in the tretinoin regimen group but were significantly increased in the NPP regimen group.

**Table 4:** Changes in stratum corneum protein analytes

	Percentage change from baseline week 8 (entire population)			Percentage change from baseline week 8 (cohorts)			Percentage change from baseline week 24 (cohorts)		
	Tretinoin regimen	NPP regimen	*P*-value	Tretinoin regimen	NPP regimen	*P*-value	Tretinoin regimen	NPP regimen	*P*-value
Soluble protein (μg mL^−1^)	140·44*	−9·43	< 0·01	160·62*	−1·83*	< 0·01	72·98*	−43·64*	< 0·01
HSA (ng mL^−1^)	52·41*	−33·01*	< 0·01	62·55*	−39·74*	< 0·01	−9·43	−73·39*	< 0·01
Involucrin (ng μg^−1^ soluble protein)	115·28*	0·46	< 0·01	155·27*	4·95	< 0·01	51·71	−31·61	< 0·05
Keratins 1, 10 and 11 (ng μg^−1^ soluble protein)	−58·79*	27·06*	< 0·01	−62·07*	18·30	< 0·01	−7·74	179·90*	< 0·01

HSA, human serum albumin; NPP, niacinamide/peptide/retinyl propionate.

* Significantly different from baseline at *P*<0·05. Listed *P*-values are for treatment comparisons.

### Cohort 24-week results

Of the 50 subjects (*n*=25 in each group) who continued on treatment for an additional 16 weeks, 48 (25, tretinoin regimen; 23, NPP regimen) completed the entire 24 weeks. As seen in Figures 2 and 4, after 8 weeks treatment the mean improvements in the two cohorts were not significantly different by either expert visual grading of the REAL images or by image analysis of the VISIA CR images. The mean cohort responses were not consistent with those determined in the full study population, as the mean tretinoin response was higher in the cohort than in the full population, while for the NPP cohort the mean response was lower. This is likely to be attributable to the self-selection of the cohort subjects, which was made prior to the start of treatment. Wrinkle severity at enrolment, however, was not significantly different between the cohorts, nor was it significantly different between the cohorts and the full populations. For both regimens, the appearance of facial fine lines and wrinkles continued to improve from 8 to 24 weeks as measured by both visual grading and image analysis (*P*<0·01 for both). Consistent with the 8-week results for the cohort, after 24 weeks of treatment there were no significant differences between the NPP regimen and the tretinoin regimen by either measure (*P*=0·74, *P*=0·77, respectively). There was a statistically significant difference in change in TEWL between the two groups at 8 and 24 weeks (*P*<0·01, Fig. 5). After 24 weeks of treatment, the stratum corneum barrier was not different from baseline in the tretinoin regimen group (*P*=0·47) but was significantly improved in the NPP regimen group (*P*<0·01).

After 16 and 24 weeks of treatment, the subset of the NPP regimen group exhibited a nonsignificant decrease from baseline in erythema and dryness (*P*>0·21, data not shown). After 16 weeks of treatment, erythema in the subset of the tretinoin regimen group was not different from baseline, but at 24 weeks of treatment there was a significant decrease in erythema. Skin dryness in the tretinoin regimen cohort was not significantly different from baseline after 16 and 24 weeks of treatment.

In general, subjects in both groups continued to notice an improvement in their skin from baseline based on the self-assessment questionnaire (data not shown). In addition, the NPP regimen subjects did not report any significant irritation after 16 and 24 weeks of treatment; the tretinoin regimen subjects did report significant peeling and flaking at 16 weeks but no significant irritation after 24 weeks (data not shown).

After 8 weeks, protein analyte changes in the cohort were consistent with those in the full population (Table 4). After 24 weeks, protein changes in both cohorts generally improved relative to 8 weeks. None the less, soluble protein levels remained significantly higher than baseline in the tretinoin regimen group and significantly lower than baseline in the NPP regimen group. Levels of HSA were similar to baseline in the tretinoin regimen group (*P*=0·59) but significantly reduced relative to baseline in the NPP regimen group. Involucrin levels remained not significantly different from baseline following 24 weeks use of the NPP regimen; however, levels of involucrin in the tretinoin regimen group decreased between 8 and 24 weeks, which is consistent with the TEWL and irritation data showing accommodation to tretinoin treatment with longer term use. Overall, the NPP regimen group had significantly lower levels of soluble protein, HSA and involucrin and significantly higher levels of keratins 1, 10 and 11 than the tretinoin regimen group at 24 weeks.

## Discussion

To our knowledge, this is the first of its kind long-term clinical study comparing a cosmetic anti-ageing regimen against a recognized prescription topical treatment for improving the appearance of facial wrinkling. The NPP regimen, which contained cosmetic ingredients known to affect the appearance of wrinkles, along with moisturizing and skin barrier building components, comparably improved the appearance of fine lines and wrinkles relative to the prescription tretinoin regimen as measured by both expert grading and image analysis in a long-term study, with better tolerability. Improvements in skin texture with topical niacinamide treatment have been reported and linked to enhanced barrier function,^7^ and a peptide used in the NPP regimen has been shown to reduce the appearance of fine lines and wrinkles without adverse effects on skin barrier.^10^ The beneficial impact of the NPP regimen ingredients is consistent with the results seen for TEWL, protein analyte changes, and the clinical data on erythema and dryness. Multiple studies have established the beneficial effects of niacinamide for improving barrier function of the stratum corneum as measured by TEWL.^18^–^20^ In the present investigation the NPP regimen had less of a positive effect on TEWL at 8 weeks vs. baseline than one would expect from the literature given that all three products contained niacinamide. The muted benefit on TEWL was probably a consequence of the wrinkle treatment product also containing retinyl propionate.

Another observation is that barrier function restoration and stabilization of stratum corneum repair mechanisms may occur at different rates, as TEWL values in the tretinoin cohort had returned to baseline by week 24 but some marker protein levels had not.

We recognize that there are limitations in this study. Tretinoin is more irritating to the skin and the products tested in the NPP regimen are more moisturizing. Additionally, while the evaluators were blinded to treatment, the subjects were not which could have affected the subjects' self-assessments. Finally, a larger 24-week cohort group size would have provided more statistical power for more robust conclusions. These limitations notwithstanding, the study data, from both objective and subjective assessments, provided consistent results and showed that the new NPP regimen improved the appearance of fine lines and wrinkles similarly to the tretinoin regimen, with fewer side-effects.

The results of this study show that the efficacy of a prescription product for improving the appearance of facial fine lines and wrinkles can be achieved with an appropriately designed cosmetic regimen, while providing additional benefits in aesthetics, skin tolerance and potential patient compliance.

## Supporting Information

**Plain English Summary S1:** Olay Professional Pro-X cosmetic anti-ageing products vs. a prescription drug, tretinoin, for treating wrinkles.

## References

[1] Nyirady J, Bergfeld W, Ellis C (2001). Tretinoin cream 0.02% for the treatment of photodamaged facial skin: a review of 2 double-blind clinical studies. Cutis.

[2] Kang S, Fisher GJ, Voorhees JJ (1997). Photoaging and topical tretinoin: therapy, pathogenesis, and prevention. Arch Dermatol.

[3] Voorhees JJ (1990). Clinical effects of long-term therapy with topical tretinoin and cellular mode of action. J Int Med Res.

[4] Weiss JS, Ellis CN, Headington JT, Voorhees JJ (1988). Topical tretinoin in the treatment of aging skin. J Am Acad Dermatol.

[5] Draelos ZD, Ertel KD, Berge CA (2006). Facilitating facial retinization through barrier improvement. Cutis.

[6] Bruce S (2008). Cosmeceuticals for the attenuation of extrinsic and intrinsic dermal aging. J Drugs Dermatol.

[7] Bissett DL, Oblong JE, Saud A (2003). Topical niacinamide provides skin aging appearance benefits while enhancing barrier function. J Clin Dermatol.

[8] Bissett DL, Miyamoto K, Sun P, Berge CA (2004). Topical niacinamide reduces yellowing, wrinkling, red blotchiness, and hyperpigmented spots in aging facial skin. Int J Cosmet Sci.

[9] Matts PJ, Oblong JE, Bissett DL (2002). A review of the range of effects of niacinamide in human skin. IFSCC Mag.

[10] Robinson LR, Fitzgerald NC, Doughty DG (2005). Topical palmitoyl pentapeptide provides improvement in photoaged human facial skin. Int J Cosmet Sci.

[11] Osborne R, Mullins LA, Jarrold BB (2008). *In vitro* skin structure benefits with a new antiaging peptide, Pal-KT. J Am Acad Dermatol.

[12] Hipkiss AR (1998). Carnosine, a protective, anti-ageing peptide?. Int J Biochem Cell Biol.

[13] Bissett DL, Mrowczynski E, Hicks S (2004). Retinyl propionate and niacinamide: reduction in excess dermal GAG’s as a mechanism for their effects in improving the appearance of aging skin. J Am Acad Dermatol.

[14] Oblong JE, Bissett DL, Draelos ZD (2005). Retinoids. Procedures in Cosmetic Dermatology, Cosmeceuticals.

[15] Johnson and Johnson (2000). *Renova®**(Generic Name: Tretinoin Cream 0.02%) Package Insert*. FDA Approved Medication Guide.

[16] Matts PJ, Miyamoto K, Hillebrand GG, Wilhelm KP, Elsner P, Berardesca E, Maibach HI (2006). Digital Imaging as an Effective Means of Recording and Measuring the Visual Signs of Skin Ageing.

[17] Hendrix SW, Miller KH, Youket TE (2007). Optimization of the skin multiple analyte profile bioanalytical method for determination of skin biomarkers from D-Squame tape samples. Skin Res Technol.

[18] Bissett DL (2002). Topical niacinamide and barrier enhancement. Cutis.

[19] Draelos ZD, Ertel K, Berge C (2005). Niacinamide-containing facial moisturizer improves skin barrier and benefits subjects with rosacea. Cutis.

[20] Crowther JM, Sieg A, Blenkiron P (2008). Measuring the effects of topical moisturizers on changes in stratum corneum thickness, water gradients and hydration *in vivo*. Br J Dermatol.

