# Supplementary material for: A randomized, controlled comparative study of the wrinkle reduction benefits of a cosmetic niacinamide/peptide/retinyl propionate product regimen vs. a prescription 0·02% tretinoin product regimen
Source: Br J Dermatol. 2010 Mar;162(3):647–54. doi: 10.1111/j.1365-2133.2009.09436.x (PMC2841824; doi:10.1111/j.1365-2133.2009.09436.x)
Supplement: Supplementary file 1 [file bjd0162-0647-SD1.pdf]

BJD
British Journal of Dermatology

Olay Professional Pro-X cosmetic anti-ageing products vs. a prescription drug, tretinoin, for treating wrinkles

With advancing age, the skin on our faces becomes increasingly wrinkled. Of significant cosmetic concern are those fine lines and wrinkles around the eye that contribute to an aged appearance. These include both fine lines under the eye as well as wrinkles that spread out from the outer corners (crow's feet).

These fine wrinkles can be reduced by regular use of cosmetic anti-ageing products or with a prescription drug called tretinoin. Because tretinoin is available only with a prescription, it is widely regarded as being more effective than cosmetic anti-ageing products. However, there is no direct scientific evidence to support this. This trial[1]—designed to address this assumption—compared a recognized topical form of tretinoin with Olay Professional Pro-X cosmetic anti-ageing products for the treatment of wrinkled and ageing skin.

How was the trial carried out?

The trial involved 196 women aged 40–65 years with moderate to moderately severe facial wrinkles. They were divided into two groups: one used 0·02% tretinoin, the other Olay cosmetic anti-ageing products (a facial cream and a targeted wrinkle treatment). All the women also used a high sun protection factor face product (SPF 30). The main purpose of the study was to compare how much the severity of wrinkles changed in the two groups after 8 weeks. The women were also asked how they felt the creams had affected their skin. Some of the women (25 in each group) initially volunteered to continue using their creams for another 16 weeks and to report again on their experiences and be technically measured after a total of 24 weeks of use.

Changes in wrinkle severity were measured from standardized high-resolution digital images by three expert graders who did not know which women were using which cream; the performance of the graders was checked by dermatologists.

What is tretinoin and what is in Olay cosmetic anti-ageing products?

Tretinoin is a retinoid (a compound related to vitamin A) prescribed to reduce the harmful effects of the sun on the skin and to reduce the appearance of wrinkles. In the trial, women using tretinoin also used Neutrogena's recommended sunscreen.

Olay cosmetic anti-ageing products contain niacinamide, peptides, carnosine and, in the case of the wrinkle treatment, retinyl propionate (a cosmetic retinoid). Women using Olay cosmetic anti-ageing products used Pro-X Age Repair Lotion as a sunscreen, which also contains vitamins C and E.

What was the main finding of the trial?

- After 8 weeks, the appearance of fine lines and wrinkles improved in both groups of women, but the change in appearance was more pronounced in those who had used the cosmetic cream regimen.

What else did it show after 8 weeks?

- Fifty-eight per cent of women using the cosmetic cream regimen 'looked better' (that is, they achieved a minimum positive grade of +1 for fewer lines and wrinkles) compared with 41% using tretinoin.
- More women had a substantial response to the cosmetic cream regimen (28% with the cosmetic vs. 13% with tretinoin).
- The cosmetic cream regimen reduced the area of eye wrinkles by a mean of 17% compared with 11% for tretinoin.
- According to the women's own assessments, the cosmetic cream regimen was better at improving the appearance and feel of their skin, and at reducing eye lines/wrinkles. They reported no difference between the regimens in their effects on fine lines/wrinkles, deep wrinkles or uneven skin texture.

What happened after 24 weeks?

The appearance of lines and wrinkles continued to improve in both sub-groups and there was no statistical difference between them after 24 weeks.

Were there any side-effects?

Tretinoin was found to have damaged the skin barrier after 8 weeks but the cosmetic cream regimen had no effect. Tretinoin also caused more skin redness and dryness than the cosmetic cream regimen, and more women using tretinoin reported itchiness and peeling or flaking of the skin.

With continued use of tretinoin, skin redness and dryness improved and, after 24 weeks, returned to their original (pre-treatment) levels. This showed that the women's skin became adjusted to the cream's effects.

What did the scientists say about their trial?

To the scientists' knowledge, this is the first long-term clinical study of its kind to compare a cosmetic anti-ageing regimen against a recognized prescription topical treatment for improving the appearance of facial wrinkling.

© 2010 The Author
Journal Compilation © 2010 British Association of Dermatologists

Facts and figures

1. The trial was conducted by researchers at Procter & Gamble in the U.S.A. in collaboration with doctors working in clinical practice, at the University of Miami and Columbia University, New York.
2. A scientific report[1] of the trial has been published in the *British Journal of Dermatology* (http://www.brjdermatol.org). This contains full details of the methods used and a statistical analysis of the results.
3. All women pre-conditioned their face for 2 weeks using (instead of their usual cosmetic skin care products) a mild skin cleanser (Olay Foaming Face Wash) and a facial moisturizer (Olay Complete All Day Moisture Lotion Sensitive Skin SPF 15) twice daily.
4. The Olay cosmetic treatment consisted of creams containing niacinamide and peptides:
   - Twice daily Olay Professional Pro-X Deep Wrinkle Treatment on the wrinkled areas; this product contains 0.3% retinyl propionate.
   - Daytime sun protection (Olay Professional Pro-X Age Repair Lotion SPF 30) in the morning and a night cream (Olay Professional Pro-X Wrinkle Smoothing Cream) in the evening, over the entire face.
5. The prescription treatment consisted of:
   - Every other evening for 2 weeks, applying 0.2% tretinoin cream (Renova) to the whole face; after 2 weeks the cream was applied every evening.
   - Daytime sun protection (Neutrogena Healthy Defense Daily Moisturiser SPF 30) every morning (sunscreen was used throughout the study).
6. Changes in the appearance of lines and wrinkles around the eyes were visually assessed before treatment and after 8 and 24 weeks using a ± 8-point scale. Changes in the area of the wrinkles were also measured by computer image analysis. Each woman assessed her treatment on a 10-point scale for several features (fine lines and wrinkles around the eyes; evenness of skin tone and texture; red blotchiness; age spots; skin firmness, radiance and hydration; and overall appearance). The effectiveness of the skin as a barrier was measured by the rate of water loss from the skin in two places on the face. Facial redness and dryness were assessed clinically by a trained expert grader using a 6-point scale.
7. The measurement that the study was designed for and which was statistically the most reliable (the 'primary endpoint') was the visually graded change in the appearance of lines and wrinkles around the eyes after 8 weeks of treatment.
8. Of the 196 women who started the trial, 97 used tretinoin and 99 used the Olay cosmetic regimen. In the tretinoin group, one woman stopped treatment after developing redness and dry skin; three others were removed from the trial because they did not use their treatment correctly. In the cosmetics group, two women were removed from the trial for not using their treatment correctly. In addition, digital imaging was not good enough to allow assessment for three women in the tretinoin group and two in the cosmetics group.

The cosmetic cream regimen contains ingredients that affect the appearance of lines and wrinkles, moisturize the skin and increase the skin barrier. Its effects on the appearance of fine lines and wrinkles were similar to that of the prescription tretinoin but it caused fewer side-effects.

The authors agreed that their trial had some limitations:

- Tretinoin is known to be more irritating to the skin whereas some of the ingredients in the cosmetic creams provide skin moisturization, so the differences in side-effects were not unexpected.
- The women using the tretinoin regimen knew which products they were using and this could have influenced their assessments.
- Finally, the number of women who used the creams for 24 weeks was relatively small, increasing the chances that some of the findings from this part of the trial may have been coincidental.

However, notwithstanding this, there was close agreement between the objective measurements from the expert graders and the subjective assessments of the women themselves.

The authors concluded that the cosmetic regimen used in this trial had effectiveness comparable with that of a prescription drug for treatment of wrinkles with added benefits for skin tolerance.

S. Chaplin
Freelance Medical Journalist, Northumberland, U.K.
E-mail: stevechaplin@btinternet.com

Conflicts of interest

S.C. is a freelance medical writer and journalist. S.C. was independently commissioned by the *British Journal of Dermatology* to write this plain-English summary, funded by Procter & Gamble. The text has been reviewed by the Journal's editors. Procter & Gamble have neither been involved in the creation nor in the editing of the summary.

Reference

1 Fu JJJ, Hillebrand G, Raleigh P, *et al.* A randomized, controlled comparative study of the wrinkle reduction benefits of a cosmetic niacinamide/peptide/retinyl propionate product regimen vs. a prescription 0.02% tretinoin product regimen. *Br J Dermatol* 2010; **162**:647–54.

© 2010 The Author
Journal Compilation © 2010 British Association of Dermatologists • *British Journal of Dermatology*
